# Supplementary material for: New species of Ontocetus (Pinnipedia: Odobenidae) from the Lower Pleistocene of the North Atlantic shows similar feeding adaptation independent to the extant walrus (Odobenus rosmarus)
Source: PeerJ. 2024 Aug 13;12:e17666. doi: 10.7717/peerj.17666 (PMC11328838; doi:10.7717/peerj.17666)
Supplement: Supplemental Information 4 — Letters of angles and measures correspond to those indicated in Boisville et al. (2022). [file peerj-12-17666-s004.docx]

|  | *Ontocetus posti* | | | *Ontocetus emmonsi* | | | | | *Odobenus rosmarus* | | | | | | | | | |
| --- | --- | --- | --- | --- | --- | --- | --- | --- | --- | --- | --- | --- | --- | --- | --- | --- | --- | --- |
|  | **NMR7472 (**♀) | **IRSNB M156 (**♂) | **RGM.St.119589 (**♀) | **IRSNB M168 (**♂) | **NMR 1890 (**♀) | **ROM 26116**  **(**♂) | **USNM PAL 374273**  **(**♀) | **USNM PAL 475482**  **(**♂) | **IRSNB 1150D (**♂) | **IRSNB 1150B (**♀) | **USNM 324983**  **(**♂) | **USNM 605099**  **(**♀) | **USNM 605100**  **(**♂) | **NMNS M27861**  **(**♀) | **NMNS M27860**  **(**♂) | **NHMUK 1948.4.24.1.**  **(**♀) | **NHMUK 1855.11.26.37**  **(**♀) | **NHMUK 1936.5.1.1 (**♂) |
| Total length of mandible | 296.8 | NA | NA | 356.2 | 209.0+ | NA | NA | NA | 247.8 | 237.9 | 296.70 | 246.34 | 323.82 | 198.84 | 250.45 | 195.87 | 186.03 | 230.0 |
| Transverse width at c1 | 67.2 | 92.0 | 66.8 | 91.6 | 31.4 | 86.8 | 48.46 | 99.94 | 78.0 | 47.6 | 98.61 | 69.19 | 105.91 | 50.57 | 76.0 | 56.02 | 58.11 | 66.33 |
| Symphysis length (anteroventral - posterodorsal) | 49.0 | 22.1 | 36.6 | 57.8 | 38.3 | 47.8+ | 42.1+ | 57.8 | 67.2 | 31.9 | 58.94 | 37.74 | 66.58 | 25.29 | 40.79 | 27.72 | 29.06 | 32.38 |
| Symphysis height (anterodorsal - posteroventral) | 86.1 | 72.6 | 79.2 | 177.0 | 105.2 | NA | NA | 156.1 | 99.7 | 80.9 | 118.65 | 76.10 | 138.40 | 60.82 | 86.03 | 60.24 | 58.11 | 84.49 |
| Ratio symphysis height / symphysis length | 1.757 | 3.285 | 2.16 | 3.062 | 2.748 | NA | NA | NA | 1.484 | 2.536 | 2.014 | 2.016 | 2.078 | 2.405 | 2.10 | 2.173 | 1.999 | 2.617 |
| Ratio symphysis height / total length of mandible | 0.290 | NA | NA | 0.497 | 0.503 | NA | NA | NA | 0.402 | 0.340 | 0.399 | 0.309 | 0.427 | 0.306 | 0.343 | 0.307 | 0.312 | 0.367 |
| Total length of the canine | 43.80 | NA | NA | 37.10 | 19.20 | NA | NA | 34.38 | 10.30 | 11.70 | NA | 14.52 | NA | 11.37 | 15.11 | 15.90 | 10.24 | NA |
| Antero-posterior diameter (APD) canine | 23.8 | NA | 18 .2 | 23.2 | 18.3 | 31.7 | NA | 33.6 | 22.1 | 17.2 | 25.53 | 19.70 | 26.67 | 16.31 | 20.49 | 17.37 | 13.57 | 17.93 |
| Transverse diameter (TD) canine | 20.0 | 34.2 | 17.3 | 20.0 | 16.8 | 32.25 | 15.6+ | 22.4 | 17.2 | 16.2 | 23.97 | 17.62 | 26.93 | 11.85 | 18.24 | 14.28 | 12.86 | 15.46 |
| APD first cheekteeth | 18.5 | 22.4 | 10.0 | 22.0 | 16.2 | 27.39 | 14.2 | 19.0 | 21.9 | 15.9 | 23.31 | 16.93 | 17.58 | 14.30 | 22.49 | 16.39 | 13.29 | 23.50 |
| TD first cheekteeth | 16.2 | 24.7 | 9.50 | 14.2 | 13.5 | 22.53 | 12.8 | 17.4 | 16.0 | 12.0 | 16.26 | 14.48 | 21.57 | 11.08 | 15.09 | 12.66 | 10.05 | 16.19 |
| APD second cheekteeth | 16.1 | 24.6 | 6.0 | 22.4 | 18.0 | 27.28 | 18.7 | 20.4 | 18.9 | 13.7 | 14.64 | 14.13 | 17.54 | 13.05 | 18.54 | 12.20 | 10.98 | 17.34 |
| TD second cheekteeth | 16.7 | 28.8 | 7.30 | 15.3 | 15.0 | 25.78 | 14.3 | 17.4 | 17.0 | 9.8 | 13.60 | 11.34 | 17.78 | 10.72 | 14.60 | 9.52 | 8.23 | 14.81 |
| APD third cheekteeth | 18.9 | 26.2 | 4.20 | 20.8 | 18.8 | 18.50 | 15.47 | 20.0 | 14.9 | 9.1 | 8.68 | 8.39 | 13.50 | 9.07 | 9.50 | 9.71 | 5.70 | 12.26 |
| TD third cheekteeth | 17.9 | 27.8 | 3.90 | 18.1 | 14.6 | 20.10 | 12.84 | 16.3 | 13.2 | 9.5 | 6.87 | 7.68 | 13.11 | 6.30 | 8.18 | 6.14 | 5.02 | 12.78 |
| APD fourth cheekteeth | 19.8 | 27.0 | NA | 16.5 | 17.0 | NA | NA | NA | x | x | x | x | x | x | x | x | x | x |
| TD fourth cheekteeth | 17.0 | 25.0 | NA | 18.6 | 13.7 | NA | NA | NA | x | x | x | x | x | x | x | x | x | x |
| APD second incisor | 9.4 | NA | NA | 11.9 | 7.1 | NA | NA | 16.5+ | x | x | x | x | x | x | x | x | x | x |
| TD second incisor | 7.7 | NA | NA | 5.3 | 6.1 | NA | NA | NA | x | x | x | x | x | x | x | x | x | x |
| APD third incisor | 14.38 | NA | 20.0 | 14.5 | 12.8 | 15.43 | NA | 20.5 | x | x | x | x | x | x | x | x | x | x |
| TD third incisor | 15.76 | NA | 14.9 | 13.2 | 14.8 | 14.02 | NA | 17.5 | x | x | x | x | x | x | x | x | x | x |
| Ratio APD cheekteeth / APD canine | 0.769 | NA | 0.366 | 0.88 | 0.945 | NA | NA | NA | 0.84 | 0.75 | 0.67 | 0.667 | 0.608 | 0.744 | 0.822 | 0.735 | 0.736 | 0.987 |
| Ratio TD cheekteeth / TD canine | 0.847 | 0.777 | 0.398 | 0.82 | 0.845 | NA | NA | NA | 0.895 | 0.644 | 0.511 | 0.634 | 0.649 | 0.79 | 0.692 | 0.661 | 0.604 | 0.944 |
| Ratio APD canine / length of dental row | 0.209 | NA | NA | 0.163 | 0.178 | NA | NA | NA | 0.263 | 0.172 | 0.265 | 0.249 | 0.290 | 0.271 | 0.253 | 0.267 | 0.225 | 0.234 |
| Length of dental row | 113.8 | NA | NA | 142.5 | 102.5 | NA | NA | NA | 84.0 | 68.0 | 96.19 | 78.87 | 91.87 | 60.08 | 80.79 | 64.97 | 60.19 | 76.51 |
| Septa between canine-first cheekteeth | 11.5 | 6.5 | 11.7 | 21.5 | 12.8 | 12.67 | 7.04 | 17.36 | 6.3 | 3.9 | 3.24 | 9.0 | 3.70 | 5.06 | 3.20 | 3.07 | 6.39 | 7.01 |
| Septa between first cheekteeth-second cheekteeth | 5.3 | 2.3 | 9.10 | 11.1 | 3.8 | 7.04 | 5.74 | 6.66 | 1.9 | 2.2 | 1.62 | 3.77 | 3.31 | 2.10 | 3.77 | 4.70 | 5.20 | 5.12 |
| Septa between second cheekteeth-third cheekteeth | 3.8 | 1.7 | 3.30 | 9.8 | 2.3 | 3.91 | 5.65 | 5.43 | 1.9 | 3.2 | 1.62 | 4.40 | 1.65 | 2.35 | 3.38 | 3.86 | 5.20 | 2.62 |
| Septa between third cheekteeth-fourth cheekteeth | 3.8 | 1.2 | NA | 6.7 | 3.5 | NA | NA | NA | x | x | x | x | x | x | x | x | x | x |
| Ratio septa between canine-first cheekteeth / length of dental row | 0.086 | NA | NA | 0.151 | 0.125 | NA | NA | NA | 0.075 | 0.057 | 0.034 | 0.114 | 0.040 | 0.084 | 0.039 | 0.047 | 0.106 | 0.092 |
| Average septa between different cheekteeth | 4.3 | 1.63 | NA | 9.2 | 3.2 | NA | NA | NA | 1.9 | 2.7 | 1.62 | 4.085 | 2.48 | 2.225 | 3.575 | 8.56 | 5.20 | 3.87 |
| Length of cheekteeth row | 78.8 | 102.9 | NA | 96.1 | 74.8 | NA | 76.5+ | NA | 57.1 | 51.2 | 62.47 | 49.40 | 60.87 | 40.63 | 55.26 | 42.37 | 40.18 | 53.07 |
| Length between last cheekteeth and vertical ramus | 17.8 | 13.1 | NA | 26.3 | 19.2 | NA | NA | NA | 20.1 | 33.9 | 32.0 | 28.50 | 36.24 | 22.95 | 19.75 | 32.76 | 39.31 | 34.88 |
| Ratio dental row length / total mandible length | 0.383 | NA | NA | 0.329 | NA | NA |  | NA | 0.339 | 0.286 | 0.324 | 0.320 | 0.284 | 0.302 | 0.322 | 0.332 | 0.323 | 0.333 |
| Ratio length between last cheekteeth and vertical ramus / total length mandible | 0.0599 | NA | NA | 0.0738 | NA | NA | NA | NA | 0.0811 | 0.1424 | 0.108 | 0.116 | 0.112 | 0.115 | 0.079 | 0.167 | 0.210 | 0.152 |
| Mandibular height (MD), after the last cheekteeth tooth | 71.1 | NA | NA | 82.0 | 56.9 | NA | NA | NA | 59.5 | 47.5 | 80.98 | 60.39 | 105.61 | 51.01 | 54.83 | 61.11 | 56.48 | 68.65 |
| Mandibular width (MT), after the last cheekteeth tooth | 32.4 | 44.7 | NA | 31.2 | 24.7 | NA | NA | NA | 35.0 | 16.7 | 41.45 | 21.38 | 43.55 | 16.93 | 32.17 | 18.29 | 14.02 | 25.60 |
| Width of the coronoid process, at its base | 17.9 | 40.0 | NA | 60.1 | 18.3 | NA | NA | NA | 22.2 | 15.0 | 26.93 | 19.51 | 37.99 | 14.26 | 25.38 | 15.36 | 13.80 | 20.41 |
| Length of the coronoid process, at its base | 92.2 | NA | NA | 63.50 | NA | NA | NA | NA | 65.90 | 58.30 | 82.78 | 72.82 | 115.09 | 63.84 | 67.56 | 83.76 | 64.04 | 91.04 |
| Ratio length of the coronoid process / total length mandible | 0.31 | NA | NA | 0.178 | NA | NA | NA | NA | 0.266 | 0.245 | 0.279 | 0.295 | 0.355 | 0.321 | 0.270 | 0.428 | 0.344 | 0.396 |
| Height of coronoid process | NA | NA | NA | 57.9 | NA | NA | NA | NA | 45.0 | 47.0 | 52.82 | 38.71 | 60.70 | 30.65 | 40.94 | 46.69 | 42.30 | 56.81 |
| Angle between anterior margin and dorsal margin (a) | 131 | NA | 166 | 159 | 146 | 139 | 143 | 152 | 151 | 152 | 155 | 146 | 145 | 147 | 153 | 153 | 146 | 143 |
| Angle between anterior margin and ventral margin (b) | 117 | NA | 134 | 122 | 137 | 137 | 131 | 113 | 141 | 135 | 141 | 146 | 134 | 129 | 141 | 151 | 137 | 139 |
| Angle between dorsal margin and ventral margin (c) | 19 | NA | 27 | 30 | 15 | 18 | 17 | 31 | 24 | 21 | 16 | 14 | 20 | 18 | 21 | 15 | 16 | 21 |
| Angle between the medial edge of mandibular condyles and the mandibular symphysis (d) | 31 | NA | NA | 16 | NA | NA | NA | NA | 23 | 23 | 22 | 24 | 22 | 28 | 25 | 26 | 21 | 23 |
| Angle between the lateral edge of the mandible and the symphysis (e) | 60 | 70 | 54 | 30 | 36 | 64 | 40 | 42 | 52 | 50 | 44 | 46 | 48 | 50 | 50 | 44 | 44 | 51 |
| Angle between horizontal and vertical ramus (f) | 132 | 142 | NA | 125 | 137 | NA | NA | NA | 136 | 127 | 129 | 128 | 122 | 117 | 124 | 129 | 118 | 129 |
| Angle between the coronoid process and the mandibular condyle (g) | 86 | NA | NA | 66 | NA | NA | NA | NA | 98 | 116 | 106 | 118 | 111 | 91 | 103 | 126 | 111 | 91 |
| Mandibular condyle width | 55.8 | NA | NA | 72.3 | NA | NA | NA | NA | 55.1 | 49.5 | 63.63 | 54.72 | 70.37 | 37.61 | 49.62 | 45.87 | 40.48 | 51.22 |
