# Supplementary figures and images for: New species of Ontocetus (Pinnipedia: Odobenidae) from the Lower Pleistocene of the North Atlantic shows similar feeding adaptation independent to the extant walrus (Odobenus rosmarus)
Source: PeerJ. 2024 Aug 13;12:e17666. doi: 10.7717/peerj.17666 (PMC11328838; doi:10.7717/peerj.17666)

A

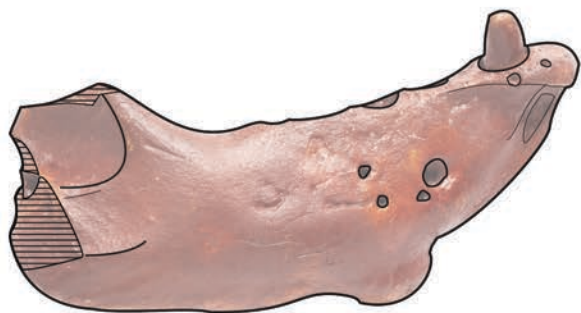

B

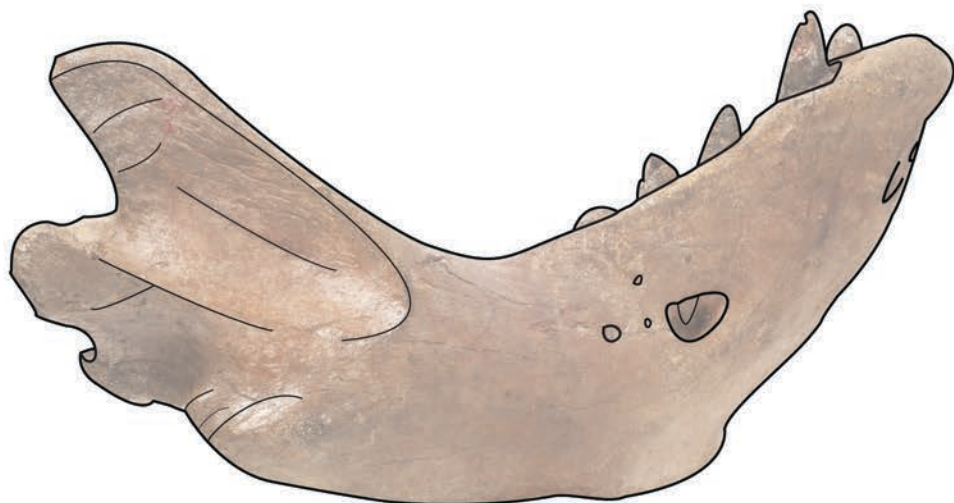

5 cm

C

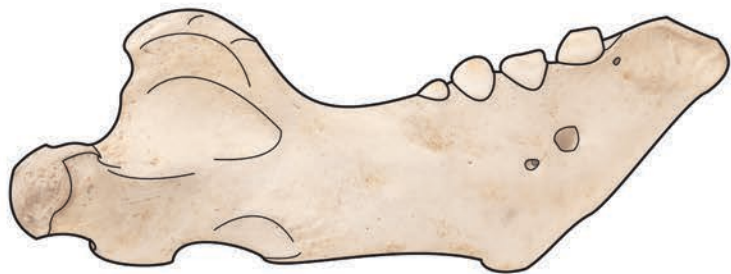

D

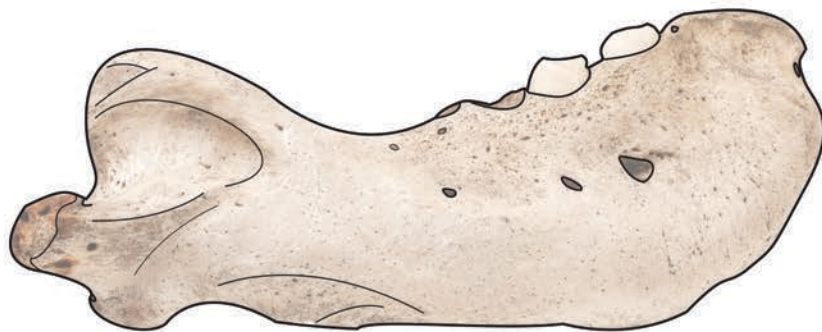

A

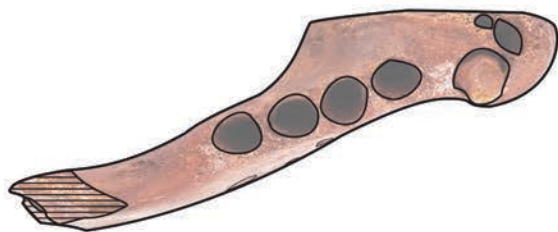

B

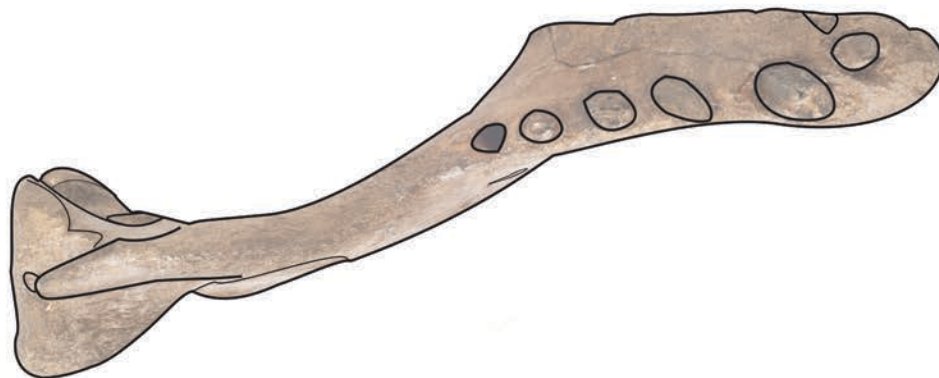

5 cm

C

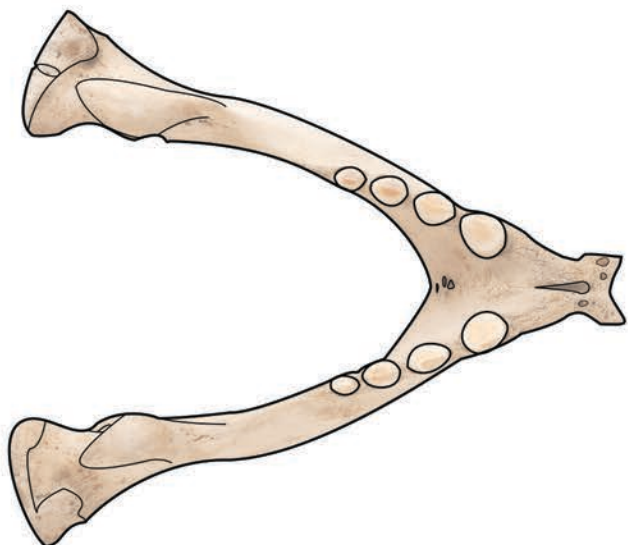

D

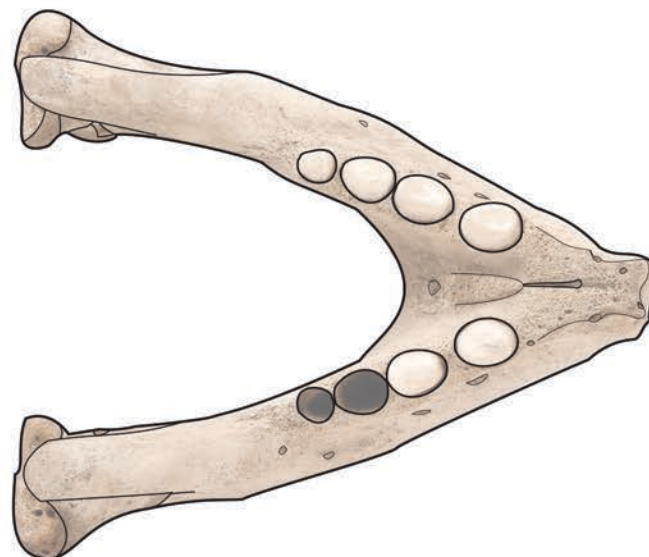

5 cm

Supplement: Supplemental Information 5 — From top to bottom: lateral view, occlusal view. Ontocetus emmonsi is represented by USNM PAL 9343 (referred female) (A) and IRSNB M168 (referred male) (B), Odobenus rosmarus is represented by IRSNB 1150B (female) (C) and IRSNB 1150D (male) (D). Scale bar equals 5 cm. Pictures taken and figures drawn by M. Boisville. [file peerj-12-17666-s005.pdf]
